# Assessment of urogenital schistosomiasis knowledge among primary and junior high school students in the Eastern Region of Ghana: A cross-sectional study

**DOI:** 10.1371/journal.pone.0218080

**Published:** 2019-06-13

**Authors:** Rachel A. Martel, Bernard Gyamfi Osei, Alexandra V. Kulinkina, Elena N. Naumova, Abdul Aziz Abdulai, David Tybor, Karen Claire Kosinski

**Affiliations:** 1 Department of Community Health, Tufts University School of Arts and Sciences, Medford, Massachusetts, United States of America; 2 University College of Agriculture and Environmental Studies, Bunso, Eastern Region, Ghana; 3 Department of Civil and Environmental Engineering, Tufts University School of Engineering, Medford, Massachusetts, United States of America; 4 Division of Nutrition Data Science, Friedman School of Nutrition Science and Policy, Tufts University, Boston, Massachusetts, United States of America; 5 Ghana Health Service, Atimpoku, Eastern Region, Ghana; 6 Department of Public Health and Community Medicine, Tufts University School of Medicine, Boston, Massachusetts, United States of America; Faculty of Science, Ain Shams University (ASU), EGYPT

## Abstract

**Background:**

Knowledge of urogenital schistosomiasis can empower individuals to limit surface water contact and participate in mass drug administration campaigns, but nothing is currently known about the schistosomiasis knowledge that schoolchildren have in Ghana. We developed and implemented a survey tool aiming to assess the knowledge of urogenital schistosomiasis (treatment, transmission, prevention, symptoms) among science teaches and primary and junior high school students in the Eastern Region of Ghana.

**Methods:**

We developed a 22-question knowledge survey tool and administered it to 875 primary and 938 junior high school students from 74 schools in 37 communities in the Eastern Region of Ghana. Teachers (n = 57) answered 20 questions matched to student questions. We compared knowledge scores (as percent of correct answers) across topics, gender, and class year and assessed associations with teacher’s knowledge scores using *t*-tests, chi-squared tests, univariate, and multivariate linear regression, respectively.

**Results:**

Students performed best when asked about symptoms (mean±SD: 76±21% correct) and prevention (mean±SD: 69±25% correct) compared with transmission (mean±SD: 50±15% correct) and treatment (mean±SD: 44±23% correct) (p<0.0005). Teachers performed best on prevention (mean±SD: 93±12% correct, p<0.0005) and poorest on treatment (mean±SD: 69±16% correct, p<0.001). When listing five facts about urogenital schistosomiasis, teachers averaged 2.9±1.2 correct. Multiple regression models suggest that gender, class year, teacher score, and town of residency explain ~27% of variability in student scores. On average, junior high school students outperformed primary school students by 10.2 percentage points (CI_95%_: 8.6–11.8); boys outperformed girls by 3.5 percentage points (CI_95%_: 2.3–4.7).

**Conclusions:**

Our survey parsed four components of student and teacher knowledge. We found strong knowledge in several realms, as well as knowledge gaps, especially on transmission and treatment. Addressing relevant gaps among students and science teachers in UGS-endemic areas may help high-risk groups recognize risky water contact activities, improve participation in mass drug administration, and spark interest in science by making it practical.

## Introduction

Schistosomiasis affects an estimated 200 million people [[1]]; over 90% of cases are in sub-Saharan Africa, and reducing this disease burden is a high priority [[2]]. Urogenital schistosomiasis (UGS), caused by *Schistosoma haematobium*, is the most common form of schistosomiasis in sub-Saharan Africa and is contracted through skin contact with surface water containing schistosome cercariae [[1], [2]]. The main risk factors for a schistosome infection are availability and use of surface water sources for domestic, recreational, and occupational purposes [[1]]. School-aged children are the demographic group most commonly affected by UGS [[1], [3]]. Estimated annual incidence among school-aged children in some towns in the Eastern Region of Ghana is 50% [[4]]; recreational swimming, being male, increasing age within adolescence, prior positive UGS status, and contact with surface water, are all possible risk factors for UGS infections in endemic locations [[4], [5]]. Control of UGS is mainly through school-based mass drug administration (MDA) with praziquantel, but MDA must be matched with complementary components such as health education and water, sanitation, and hygiene improvements [[6], [7], [8]].

In the Middle East and sub-Saharan Africa, schistosomiasis (*S*. *haematobium* and *S*. *mansoni*) knowledge among schoolchildren and the general population is poor, with misconceptions about transmission, treatment, symptoms, and prevention. For example, many people do not associate transmission with water contact, instead believing that transmission routes include drinking contaminated water [[9], [10], [11], [12], [13]], breathing contaminated air, using a contaminated latrine [[14]], eating contaminated food [[12], [13], [14]], contact with soil [[13], [14]], sexual contact [[15], [16], [17]], and witchcraft [[15], [17], [18]].

Educational programs have the potential to ensure basic knowledge of UGS, which is an important step towards empowering individuals in endemic areas to take precautions, such as limiting surface water contact and participating in regular MDA campaigns, and to reduce stigmatization and taboos. The World Health Organization (WHO) [[19]] has emphasized school-based instruction about UGS, and has developed educational materials with information about the schistosome life cycle, symptoms, treatment, and health-seeking behavior. Knowledge of UGS should be assessed before and after any UGS health education program is undertaken to determine whether the instruction effectively imparts knowledge. However, no tools exist to systematically assess UGS knowledge, so education programs cannot easily be evaluated or compared with each other. Previous research has shown positive behavior changes for some demographic groups following improvements in schistosomiasis knowledge [[20], [21], [22]], but none of these studies used a standardized tool for knowledge assessment, so they cannot be directly compared with each other. To the best of our knowledge, there is also no current published literature on the knowledge of UGS among schoolchildren and their teachers in Ghana.

Our study objective was to assess student and teacher UGS knowledge in the Eastern Region of Ghana across four categories: transmission, treatment, prevention, and symptoms. We designed a 22-question knowledge survey tool based on factual information about schistosomiasis found in the peer-reviewed literature and (WHO) educational materials [[19]]. We examined whether the percent of correct responses varied across gender, class year (primary versus junior high school), town of residence, and whether they were associated with teacher knowledge. We recommend that this survey tool be used as-is or adapted to local settings in sub-Saharan Africa to assess existing UGS knowledge.

## Methods

### Study design and recruitment

We chose the Eastern Region of Ghana because UGS is endemic [[23]] and surface water is widely available and used [[24], [25], [26], [27]]. The Eastern Region covers 19,323 km^2^ with topography ranging from low valleys to some of the highest peaks in the country ([28]). Vegetation is tropical, rainfall peaks twice per year, and agriculture and animal husbandry are widely practiced, with agriculture being the primary economic activity ([28]). As of the 2010 Census, there were 2,633,154 people in this region; Akans are the dominant ethic group, but the region is very ethnically and culturally diverse due to migration ([28]).

**Fig 1:**
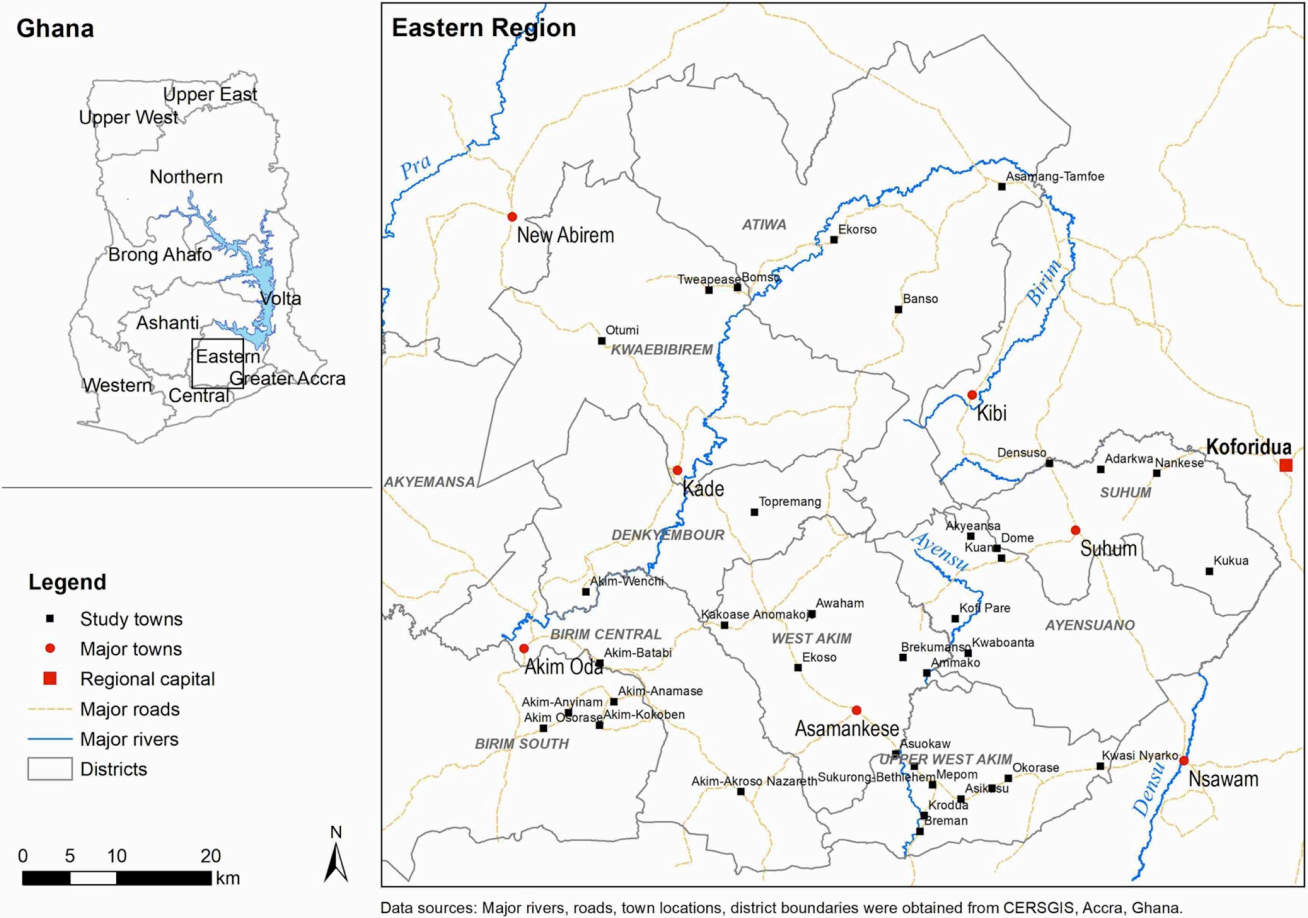
Map of the Eastern Region of Ghana showing study towns, other major towns, and the regional capital.

For our cross-sectional study, we took a simple random sample of 40 rural communities (population range 500–5,000) from a sampling frame of 74 communities developed for another study [[26]] (Fig 1). Given limited time and financial resources, we chose the largest public primary school and junior high school in each town. Because we were not seeking to test a specific hypothesis, we did not perform a priori power calculations; instead, sample size was driven by the amount of resources available, and we believe it was large enough to provide estimates with sufficient precision to answer our aims about assessing student and teacher knowledge. All fourth-year primary school (P4) students (~11 years, n = 875) and second-year junior high school (JHS2) students (~15 years, n = 938) were invited to participate in a study about “bloody pee”/“*dwensɔ mogya*”. We selected P4 students because they were the youngest students consistently able to follow along with the survey questions, and they are at high risk of UGS [[28], [29]]. As a comparison, we asked JHS2 students to participate because they were the oldest students available at the time of the survey and were expected to represent an accumulation of knowledge over time. Students’ respective science teachers responsible for health education (n = 57) were also invited to participate.

The Institutional Review Board of Tufts University in Medford, Massachusetts approved this minimal risk study as exempt, Categories 1 and 2. Given the exempt status of the study, informed consent, parental consent, and a waiver of informed consent were not required. However, to address any potential ethical concerns, all participants were informed that the study was anonymous and that they could opt out at any time. Head/assistant head teachers gave verbal permission for schools to participate. All individuals who were invited to participate in the study chose to do so.

### Data collection

#### Student knowledge data

We designed the written survey based on factual information in the peer-reviewed literature and (WHO) [[19]] educational materials (S1 Appendix). We also based survey questions on our experience in endemic areas and interactions with Ghanaian health professionals. Twenty-one questions in the final survey tool were closed-ended (“True”, “False”, or “I don’t know”) and one was open-ended about UGS treatment. The “I don’t know” option was included to reduce guessing. Questions were in four areas: transmission (9 questions), prevention (4 questions), symptoms (6 questions), and treatment (3 questions). The expectation was not that students should be able to achieve 100%, but rather, that a variety of different questions would provide information about existing knowledge in 4 realms, and that associations with student knowledge could be explored among a range of scores.

The survey tool was written in English by two native English-speakers, translated to Twi by a different individual who is a native Twi-speaker, back-translated to English by a fourth individual, and pilot tested in non-participating schools. Following pre-testing, we removed slightly more than half of the questions to reduce the overall time required for the survey; the questions we removed were mainly those assessing internal validity and most were redundant with others that we retained. We added one additional question for which there was no corollary on the teacher survey tool due to an oversight. We also slightly revised the language of several questions to make them clearer, which resulted in one question no longer aligning directly with a question on the teacher survey tool. In addition to the 22 knowledge questions, students were asked to mark their gender, age, and class year. Data were collected (June 15^th^-July 1^st^ 2015) during school hours in classrooms. Questions were written in English, the official language of school instruction and of school textbooks, and read aloud multiple times in Twi by a native speaker to ensure understanding. While there are no documented differences in English/Twi literacy in the Eastern Region [[28]], reading questions aloud in Twi was used to overcome any reading difficulties that students may have had in English; Twi is sometimes used verbally by teachers in the Eastern Region to provide additional clarification.

#### Teacher knowledge data

The written survey tool that we created for the teachers was extremely similar to the survey tool we originally developed for schoolchildren so that student and teacher questions could be matched after data collection (S2 Appendix). The survey tool for teachers was written in English by two native English-speakers and pilot tested with teachers in non-participating schools. Following pre-testing, we retained all 52 of the original questions and made a few minor wording changes for clarity. Closed-ended questions for the teachers did not include the “I don’t know” option.

The survey was administered in English. It first asked teachers to list 5 facts about UGS (open-ended) to assess knowledge without recognition bias. The listing exercise required retrieval of facts and likely brought forward only those pieces of information that are most salient. Two study team members independently coded each response as correct (1 point), incorrect (0 points), or missing (0 points); discrepancies were discussed and rectified. “Missing” included listing fewer than 5 facts, repetition, or non-specificity. After the listing question, teachers answered 2 open-ended questions about treatment, 48 closed-ended questions, and one additional open-ended question about their guidance to students regarding the best ways to protect one’s self from UGS. Of these original 52 questions, 20 were matched with questions from the student survey and used in the present analysis, and the listing question data was also analyzed here. The remaining 31 questions will be analyzed separately.

### Data analysis

We assessed student UGS knowledge by estimating their scores, e.g. summing correct answers and converting to percentages for each of four categories, as well as overall percentages. The four categories were transmission, treatment, prevention, and symptoms. For our second objective, we compared the percent of correct answers and the frequency of “I don’t know” responses for boys and girls, and also for primary and junior high school students. We examined the distributions of knowledge scores with respect to the presence of outliers, skewness, and degree of departure from normality assumptions. While the standard Kolmogorov-Smirnov and Shapiro-Wilks tests indicated a departure from normality (S1 Table), the distributions appeared to be symmetrical with no apparent outliers (S1 Fig). Given the overall sample size of 1,813 respondents, we proceeded with the standard *t*-test to compare scores in the 4 survey categories and chi-square tests to compare the frequencies across class year, gender, and location. We conducted all analyses in SPSS (Version 23.0.0.0) with a two-sided alpha level of 0.05, when relevant.

For teacher knowledge, we gave one point for each of five listed facts about UGS and for each correct answer of 20 survey questions. We calculated separate scores for each of the 4 categories that matched student categories. For validation purposes, we applied a paired *t*-test to examine whether performance on the listing section differed from teacher performance on the 20-question survey.

We then further assessed associations between students’ knowledge score and gender, class year, town/district, and teacher knowledge using univariate and multivariate linear regression models. We calculated Spearman correlation coefficients (2-tailed, alpha = 0.01) between overall teacher and student scores to capture the linear or monotonic relationships. To determine whether teacher scores were associated with student scores when controlling for gender and class, we included overall teacher scores in univariate and multivariate models. We then added information about location to the multivariate model to adjust for spatial variability in student scores.

For all survey questions, we originally planned to test for internal validity by asking repeated questions with wording variations throughout the survey. However, when we piloted this form of the survey, the length with the repeated questions made it inappropriately long for the student ages in our study and we removed those questions. Instead, we used expert validity and face validity for initial question development, and also determined during data analysis whether the relatively likelihood of correct responses was higher with questions that were expected to be easier.

## Results

### Urogenital schistosomiasis knowledge: schoolchildren

Schools in 37 of the 40 selected communities participated; in 3 communities, school was not in session on the day the school was approached for participation. A total of 1,813 schoolchildren participated (n = 875 P4, mean age: 11.5±1.5, n = 938 JHS2, mean age: 15.5±1.5) (Table 1; S2 and S3 Tables). Fifty-one percent of P4 and 45% of JHS2 students were female. P4 overall scores (51.4±13.5%) were significantly lower than JHS2 overall scores (63.0±10.2%) (p<0.0005) (Table 1). These class year differences persisted when we stratified data by district and town (S2 and S3 Tables). In comparing all towns to the lowest-performing town, there was also a significant difference in performance between students across towns, which was expected (p<0.0005).

**Table 1:** Scores on a 22-question UGS knowledge survey for schoolchildren in the Eastern Region, Ghana.

Town	Class	n	Mean Total Score (SD)	Mean Transmission Score (SD)	Mean Treatment Score (SD)	Mean Protection Score (SD)	Mean Symptoms Score (SD)
Adarkwa	JHS 2	14	61.0 (11.1)	52.0 (12.6)	47.6 (21.5)	82.1 (18.2)	75.0 (19.6)
	P4	22	37.8 (18.5)	31.4 (19.8)	24.2 (25.6)	44.3 (29.8)	59.1 (23.8)
Akim Akroso	JHS 2	16	67.6 (10.3)	65.3 (12.1)	43.8 (23.5)	71.9 (25.6)	87.5 (12.9)
	P4	8	48.9 (10.8)	40.9 (13.7)	33.3 (17.8)	59.4 (26.5)	71.9 (20.9)
Akim-Anamase	JHS 2	15	60.0 (5.7)	52.1 (12.1)	40.0 (18.7)	76.7 (20.0)	80.0 (14.0)
	P4	19	62.0 (10.8)	59.8 (16.7)	28.1 (22.9)	80.3 (19.7)	75.0 (16.7)
Akim-Anyinam	JHS 2	19	57.4 (9.6)	45.5 (11.3)	42.1 (15.1)	77.6 (14.2)	81.6 (20.1)
	P4	17	49.5 (11.7)	41.2 (12.9)	47.1 (20.6)	47.1 (26.3)	76.5 (16.5)
Akim-Batabi	JHS 2	33	67.4 (5.7)	52.6 (8.4)	66.7 (0.0)	83.3 (13.5)	92.4 (11.7)
	P4	12	56.8 (8.6)	53.8 (12.5)	47.2 (22.3)	54.2 (23.4)	75.0 (21.3)
Akim-Kokoben	JHS 2	30	64.8 (8.0)	51.2 (11.1)	56.7 (15.5)	87.5 (15.7)	85.8 (12.6)
	P4	28	55.5 (14.1)	48.1 (14.0)	53.6 (21.0)	64.3 (24.9)	68.8 (24.2)
Akim-Osorase	JHS 2	31	63.0 (8.7)	52.8 (9.5)	50.5 (19.0)	79.0 (19.5)	84.7 (14.0)
	P4	26	56.3 (8.4)	46.9 (16.2)	47.4 (16.8)	66.4 (22.3)	78.9 (11.6)
Akim-Wenchi	JHS 2	27	63.1 (9.5)	54.9 (10.5)	49.4 (19.3)	77.8 (18.8)	81.5 (13.1)
	P4	24	54.2 (13.9)	45.8 (18.5)	52.8 (16.8)	67.7 (15.6)	64.6 (23.2)
Akyeansa	JHS 2	28	53.9 (10.8)	45.1 (13.0)	48.8 (19.2)	63.4 (17.3)	72.3 (27.5)
	P4	14	40.9 (14.7)	34.6 (16.7)	33.3 (22.6)	42.9 (22.8)	57.1 (22.8)
Ammako	JHS 2	30	56.7 (9.8)	44.6 (12.7)	54.4 (16.3)	68.3 (24.5)	80.0 (16.6)
	P4	18	58.1 (8.5)	44.4 (12.8)	48.2 (20.5)	75.0 (22.7)	86.1 (12.8)
Asamang Tamfoe	JHS 2	15	65.8 (10.6)	57.0 (12.6)	53.3 (16.9)	85.0 (18.4)	80.0 (19.4)
	P4	28	56.5 (11.7)	50.7 (16.10	42.9 (23.8)	60.7 (20.9)	78.6 (18.9)
Asikasu	JHS 2	27	70.5 (6.8)	65.7 (11.6)	59.3 (14.1)	82.4 (16.7)	80.6 (17.5)
	P4	33	53.9 (12.6)	48.8 (14.5)	45.5 (16.3)	61.4 (25.8)	66.7 (21.3)
Asoukaw	JHS 2	49	64.2 (11.3)	56.4 (13.3)	42.2 (21.3)	78.6 (21.0)	87.8 (16.2)
	P4	52	46.3 (14.7)	46.2 (15.2)	18.6 (24.2)	56.3 (32.0)	57.7 (24.5)
Asuotwene	JHS 2	24	58.7 (9.8)	50.8 (13.9)	54.2 (19.2)	72.9 (14.6)	69.8 (20.8)
	P4	24	52.1 (12.7)	45.5 (15.4)	34.7 (20.8)	59.4 (29.3)	76.0 (17.3)
Awaham	JHS 2	28	67.5 (7.7)	59.7 (10.3)	51.2 (19.2)	83.0 (13.7)	85.7 (20.9)
	P4	48	54.6 (12.7)	48.9 (14.50	43.1 (24.8)	68.8 (22.8)	65.1 (24.6)
Banso	JHS 2	24	69.3 (7.5)	59.9 (8.4)	59.7 (17.0)	81.3 (11.1)	90.6 (16.2)
	P4	17	62.0 (14.4)	57.2 (16.6)	52.9 (20.6)	72.1 (23.2)	72.1 (17.4)
Bomso	JHS 2	45	62.3 (7.2)	51.5 (9.7)	48.9 (18.3)	79.4 (17.9)	85.0 (17.2)
	P4	20	54.3 (12.7)	48.6 (16.2)	33.3 (24.2)	66.3 (28.4)	73.8 (15.1)
Brekumanso	JHS 2	43	58.5 (11.3)	49.3 (14.2)	51.9 (18.3)	72.1 (26.8)	75.0 (15.4)
	P4	18	48.5 (13.9)	45.5 (19.2)	38.9 (23.6)	41.7 (25.7)	70.8 (23.1)
Breman	JHS 2	7	58.4 (7.2)	44.2 (9.7)	38.1 (23.0)	89.3 (13.4)	82.1 (12.2)
	P4	12	50.8 (10.6)	40.9 (9.9)	47.2 (22.3)	52.1 (24.9)	79.2 (14.4)
Densuso	JHS 2	8	65.9 (6.4)	56.8 (6.4)	58.3 (15.4)	75.0 (0.0)	87.5 (18.9)
	P4	15	54.8 (5.8)	48.5 (10.7)	42.2 (19.8)	68.3 (17.6)	68.3 (25.8)
Dome	JHS 2	20	58.2 (8.5)	50.9 (9.5)	43.3 (24.4)	72.5 (19.7)	75.0 (14.0)
	P4	23	46.6 (10.1)	38.7 (12.0)	27.5 (19.2)	60.9 (27.0)	68.5 (18.8)
Ekorso	JHS 2	12	61.4 (10.5)	52.3 (18.6)	52.8 (17.2)	75.0 (18.5)	79.2 (17.9)
	P4	8	51.1 (12.3)	44.3 (12.3)	50.0 (25.2)	53.1 (28.2)	68.8 (32.0)
Ekoso	JHS 2	42	68.2 (8.1)	61.7 (13.7)	37.3 (16.8)	91.7 (14.3)	85.7 (13.7)
	P4	74	47.4 (14.8)	42.8 (17.5)	26.6 (19.9)	58.5 (23.9)	64.9 (26.1)
Kakoase Anomakojo	JHS 2	10	59.5 (10.2)	48.2 (13.6)	56.7 (22.5)	72.5 (18.4)	80.0 (15.8)
	P4	22	57.2 (10.1)	50.8 (16.3)	47.0 (19.7)	68.2 (24.6)	71.6 (11.7)
Kofi Pare	JHS 2	11	59.9 (9.0)	47.9 (13.5)	54.6 (16.8)	77.3 (20.8)	79.6 (18.8)
	P4	13	49.7 (9.0)	41.3 (10.9)	33.3 (23.6)	59.6 (19.2)	75.0 (10.2)
Krodua	JHS 2	24	59.8 (10.6)	48.1 (14.6)	51.4 (19.6)	80.2 (18.0)	78.1 (11.2)
	P4	19	56.2 (10.7)	52.6 (13.4)	52.6 (16.9)	54.0 (24.0)	71.1 (25.4)
Kuano	JHS 2	20	64.3 (10.7)	54.6 (12.2)	53.3 (16.8)	81.3 (13.8)	82.5 (18.3)
	P4	19	51.0 (15.0)	43.1 (18.6)	35.1 (23.5)	68.4 (27.4)	67.1 (20.5)
Kukua	JHS 2	15	55.2 (6.8)	44.9 (9.4)	46.7 (21.1)	63.3 (22.9)	81.7 (11.4)
	P4	17	48.4 (11.1)	42.8 (14.3)	33.3 (23.6)	45.6 (20.2)	77.9 (15.0)
Kwaboanta	JHS 2	23	68.8 (6.8)	61.3 (11.4)	43.5 (21.2)	85.9 (16.6)	91.3 (12.2)
	P4	20	53.6 (16.6)	56.4 (18.1)	35.0 (22.9)	43.8 (24.2)	70.0 (17.4)
Kwasi Nyarko	JHS 2	20	65.2 (10.0)	56.8 (13.1)	45.0 (22.4)	81.3 (16.0)	87.5 (15.2)
	P4	34	52.3 (15.8)	54.6 (17.5)	22.6 (19.6)	58.8 (28.8)	61.8 (26.3)
Mepom	JHS 2	46	68.4 (11.0)	58.9 (14.7)	69.6 (9.5)	81.0 (21.8)	81.0 (18.4)
	P4	36	45.7 (14.3)	40.7 (14.7)	38.9 (21.8)	49.3 (33.5)	61.1 (24.2)
Nankese	JHS 2	16	60.2 (11.4)	51.1 (12.4)	52.1 (17.1)	75.0 (28.9)	76.6 (17.0)
	P4	12	43.6 (12.0)	37.1 (15.2)	33.3 (20.1)	52.1 (24.9)	60.4 (19.8)
Okorase	JHS 2	49	60.3 (8.9)	49.9 (11.2)	50.3 (21.6)	74.5 (18.7)	82.1 (16.1)
	P4	30	56.7 (8.8)	48.2 (12.7)	45.6 (18.5)	68.3 (21.7)	76.7 (13.0)
Otumi	JHS 2	39	65.2 (11.9)	56.9 (15.4)	54.7 (16.2)	83.3 (20.1)	77.6 (20.5)
	P4	33	46.4 (11.0)	42.2 (12.6)	28.3 (16.9)	48.5 (27.2)	69.7 (18.5)
Sukurong Bethlehem	JHS 2	26	62.9 (13.0)	54.2 (14.7)	52.6 (21.4)	80.8 (26.7)	76.9 (18.6)
	P4	18	50.3 (9.7)	43.9 (11.4)	42.6 (27.5)	56.9 (24.0)	66.7 (21.0)
Topremang	JHS 2	23	64.0 (7.5)	52.6 (12.5)	49.3 (17.0)	81.5 (11.2)	89.1 (12.7)
	P4	17	52.1 (11.1)	44.9 (10.9)	37.3 (20.0)	60.3 (28.0)	75.0 (17.7)
Tweapease	JHS 2	29	60.2 (10.3)	53.0 (11.7)	43.7 (22.0)	71.6 (20.8)	81.0 (17.2)
	P4	25	50.9 (10.7)	45.1 (15.2)	36.0 (25.3)	59.0 (24.9)	70.0 (17.7)
**Totals**	**All**	**1813**	**57.4 (13.2)**	**50.0 (15.1)**	**44.4 (22.5)**	**69.2 (25.1)**	**75.8 (20.6)**
	**JHS 2**	**938**	**63.0 (10.2)**	**53.7 (13.2)**	**51.1 (19.5)**	**78.6 (19.8)**	**82.1 (17.3)**
** **	**P4**	**875**	**51.4 (13.5)**	**46.1 (16.0)**	**37.3 (23.3)**	**59.2 (26.4)**	**69.0 (21.7)**

**Fig 2:**
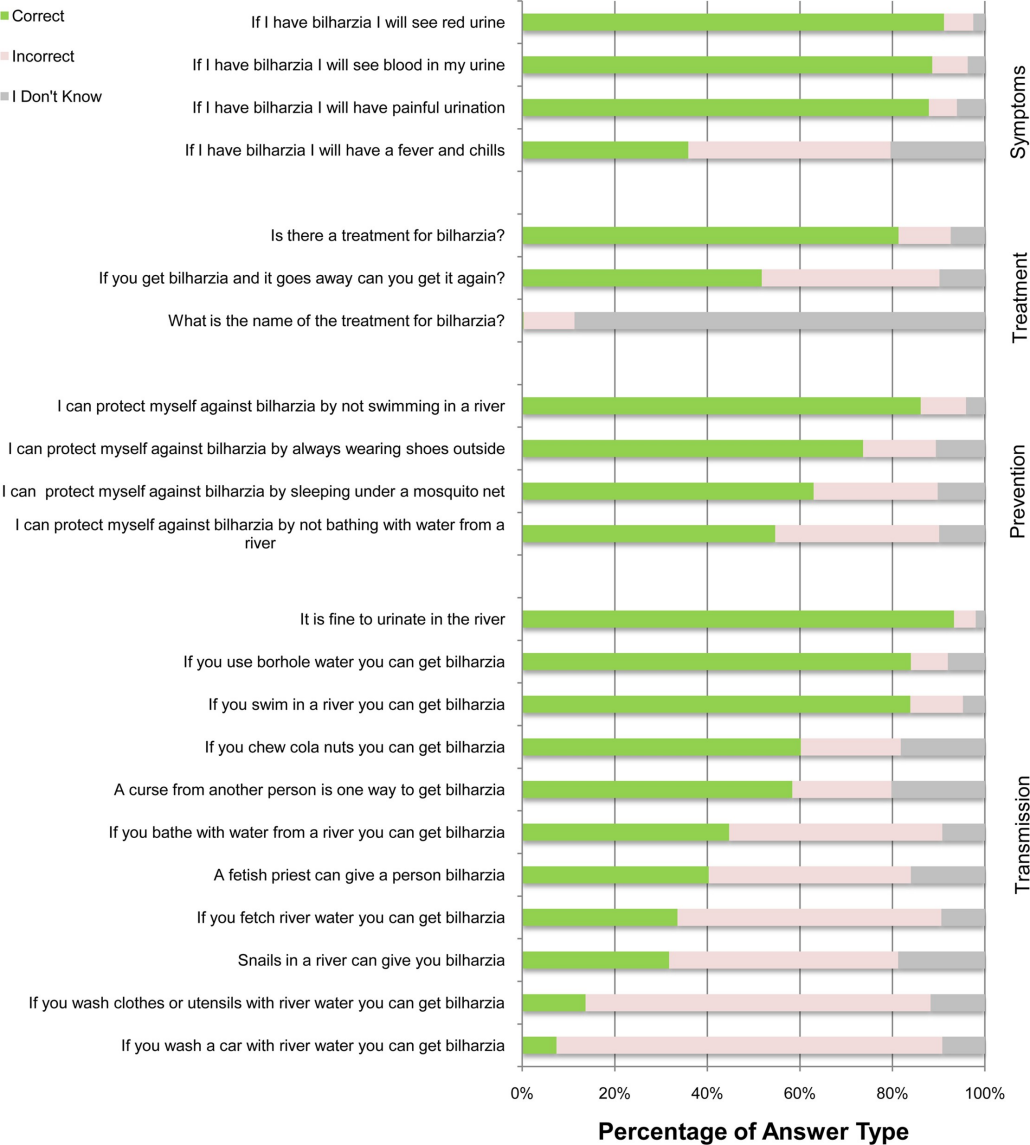
The distribution of responses to a 22-question UGS knowledge survey given to 1,813 schoolchildren from 37 towns in the Eastern Region of Ghana. 875 students were P4 and 938 were JHS2; coding: 1 = transmission, 2 = treatment, 3 = protective measures, and 4 = symptoms.

Students performed better with symptoms and protective measures (mean scores: 75.8±20.6% and 69.2±25.1%, respectively), compared with questions about treatment and transmission (mean scores: 44.4±22.5% and 50.0±15.1%, respectively). Students expressed the most uncertainty about UGS treatment (Fig 2). Only 0.3% of students (all JHS2), answered this question correctly; 88.7% of students answered, “I don’t know” and others responded incorrectly. Students were aware that urinating in rivers can transmit *S*. *haematobium* (JHS2: 96.5% correct; P4: 89.8% correct), yet most students wrongly assumed that car-washing with surface water would not facilitate the transmission (JHS2: 5.2% correct; P4: 9.7% correct) (Fig 2).

Boys (60±12.4%) scored significantly higher than girls (55±13.4%) (p<0.0005), even after stratifying by district (S2 Table); this gender difference was more pronounced for P4 than JHS2 students (S2 Fig).

### Urogenital schistosomiasis knowledge: teachers

We located 57 (30 JHS2, 27 P4) of the 74 teachers on the day we visited each school (7 JHS2 teachers and 10 P4 teachers were missing). For the open-ended listing exercise, teachers named an average of 2.9±1.2 correct UGS facts (JHS2: 3.0±1.1 versus P4: 2.7±1.3, p = 0.25), 0.67±0.79 incorrect facts, and left 1.5±1.3 spaces blank. The two most common correct responses were that urinating blood is a symptom of UGS, and that UGS is a waterborne or water-related disease. While a waterborne disease is defined as a disease transmitted through drinking water, we scored this response correct as it is probable that teachers did not use the term in its technical sense, but rather to describe the presence of water in the transmission cycle of UGS. The answer that UGS is a “river-borne” disease or a disease that one gets from rivers and ponds was also included in this category. The two most common incorrect responses were that transmission occurs via drinking unclean water, and the causative organism is a bacterium (Table 2).

**Table 2:** The most common correct and incorrect responses to a listing question asking teachers for 5 facts about UGS.

**Correct Responses**	**Frequency**
Urinating blood is a symptom	42
Water borne/related disease	30
Contract it by swimming/bathing in surface water	18
Painful urination is a symptom	12
Causative organism lives in snails	8
Can be cured	6
Caused by worms	6
Skin itching is a symptom	6
Abdominal pain is a symptom	4
Can be treated	4
Can cause infertility	4
Caused by a blood fluke	3
**Incorrect Responses**	** **
Contracted by drinking unclean water	6
Caused by bacteria	5

**Fig 3:**
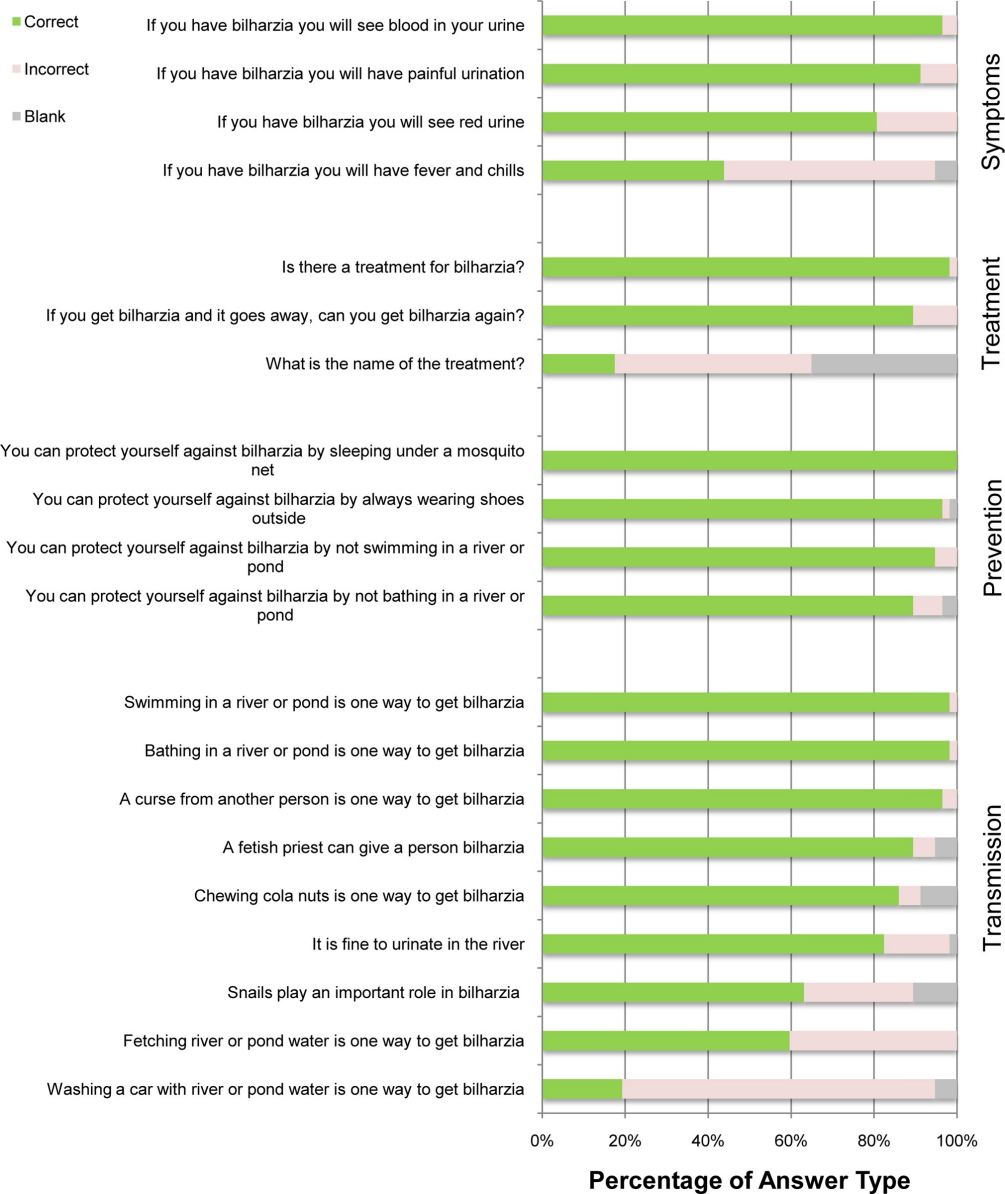
Responses to a 20-question UGS knowledge survey administered to teachers (n = 30 JHS2 and n = 20 P4) in the Eastern Region of Ghana; coding: 1 = transmission, 2 = treatment, 3 = protective measures, and 4 = symptoms.

Teachers performed significantly better on the 20 closed-ended questions than they did on the listing exercise (p<0.005). Teachers answered 80±8.0% of 20 UGS knowledge questions correctly (JHS2: 83±7.0%; P4: 77±8.0%, p = 0.004). While 56 of 57 teachers knew that UGS is treatable, only 10 (17.5%) knew the treatment name. Questions about treatment and whether transmission occurs during car washing were the most difficult for teachers, which matches with responses given by students. Teachers performed best on questions about whether mosquito nets protect against UGS (Question 23) (100% correct), the existence of treatment (Question 2) (98% correct), and transmission via swimming (Question 13) (98% correct) and bathing (Question 9) (98% correct) (Fig 3).

The open-ended listing exercise and the closed-ended questions for teachers assessed two different types of knowledge and the two were not significantly correlated (*r*_*s*_ = 0.098, α = 0.469). However, the same teachers scored well (80±8.0%) on the 20 closed-ended questions.

### Significant associations with student knowledge

**Fig 4:**
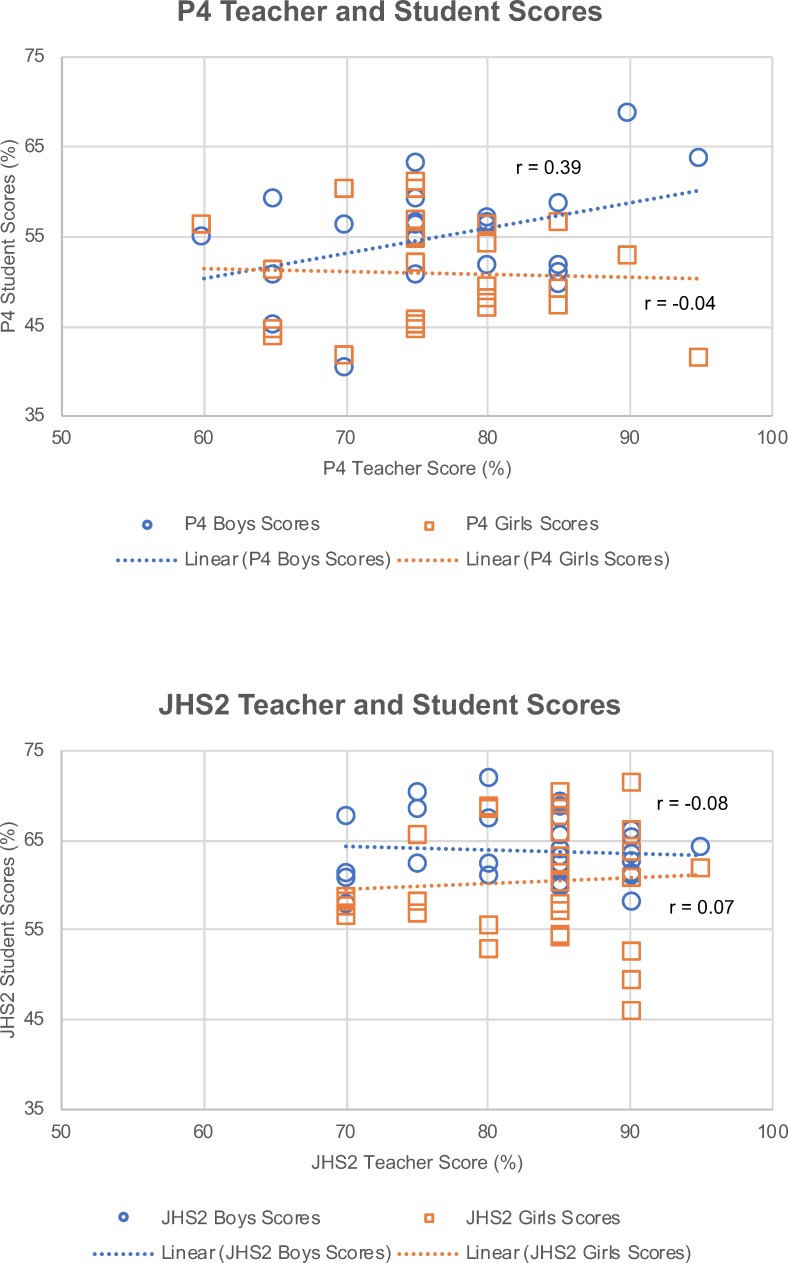
Scatterplots showing the relationship between teacher scores and mean student scores for P4 (a) and JHS2 classrooms (b), stratified by student gender.

Scatterplots of scores by class year and gender show a weak association between P4 boys and P4 teachers (r = 0.39), but not between other pairings (Fig 4A and 4B). Univariate linear regression models suggest that gender, class year, district, and town are significantly associated with student scores (S4 and S5 Tables). Teacher score on the listing exercise is not significant (r = 0.05, p = 0.057) (S5 Table, model 5), while teacher score on the 20 matched questions is significantly associated with student scores (S5 Table, model 6) (r = 0.23, p<0.0005). Once the univariate model of teacher score versus student score is split by class year (P4 and JHS2), teacher score is significant only for P4 students and not for JHS2 (r = 0.12, p = 0.002 and r = 0.054, p = 0.131, respectively) (S5 Table, models 7 and 8).

A multivariate regression model with gender, class year, and teacher score explains 20.0% of variability in student scores; this same model adjusted for town explains 27% of the variability but adjusting for town has a very limited impact on model coefficients (Table 3). In the unadjusted model, class year effect is greatest, with JHS2 outperforming P4 students by 9.6 percentage points (CI_95%_: 8.3–10.9). Boys outperformed girls by 3.6 percentage points (CI_95%_: 2.4–4.8). An improvement in teacher score by about 8 additional correct questions out of 20 improves student scores by 1 answer on the 22-question survey.

**Table 3:** Multivariate linear regression models with student scores on a 22-question UGS knowledge survey as the dependent variable and with gender, class year, and teacher score as independent variables, adjusted and unadjusted for town.

	Model Not Adjusted for Town (n = 1,813)			Model Adjusted for Town (n = 1,693)		
	B	Sig.	95.0% CI	B	Sig.	95.0% CI
Gender (Ref = Girls)	3.6	**<0.0005**	(2.4, 4.8)	3.5	**<0.001**	(2.3, 4.7)
Class Year (Ref = P4)	9.6	**<0.0005**	(8.3, 10.9)	10.2	**<0.001**	(8.6, 11.8)
Teacher score (20 questions)	0.13	**0.001**	(0.06, 0.2)	0.13	**0.03**	(0.01, 0.26)

## Discussion

### Key results

Our survey tool assessed factual knowledge of UGS among schoolchildren and teachers in 4 key areas. Gender, class year, and teacher knowledge were significantly correlated with student knowledge in a multivariate model. For students, the survey showed that JHS2 students outperformed P4 students, and that boys performed significantly better than girls. Girls were significantly more likely than boys to choose “I don’t know” on 14 of 22 questions (chi-square, p<0.05). Our survey also showed that student participants recognized important UGS symptoms, as well as disease risk posed by swimming in surface water, but they considered other water contact activities less risky. Most participants were aware that UGS treatment exists, but very few could name praziquantel. We also assessed teacher knowledge as it related to student knowledge. In the listing exercise, 30 teachers noted a relationship between UGS and water, and 18 wrote that swimming is a risk factor, but none named skin contact with water as the transmission method. Teachers generally did well on the closed-ended questions, showing that they were mostly able to recognize correct information and likely have past familiarity with UGS.

### Limitations

Our study has several limitations. We surveyed students in two schools per town (one primary and one JHS); it would be ideal to consider multiple schools within the same community to determine whether there are statistically significant differences. Additional work could be done to ensure internal and external validity of the survey tool. We originally developed the survey with a number of additional questions to assess internal validity, but this caused the survey to be far too long for children.

### Interpretation

We identified several notable patterns in student knowledge. We found that JHS2 students performed better than P4 students, which was expected both because JHS2 students are older, and thus better able to solve problems and think analytically, but also because they have received a greater amount of instructional time devoted to UGS. Other studies have shown similar findings [[30], [31]]. We saw gender-based differences in student responses as well. In our study area, boys are more likely to have UGS [[1]], so they may recognize aspects through experience. Girls were significantly more likely than boys to choose “I don’t know”. This demonstrates the value of including an “I don’t know” response, which can reduce guessing and provide additional information about confidence in one’s responses.

Gender-based differences in knowledge have been shown elsewhere. Mwai et al. [[30]] in Kenya found no difference in UGS knowledge between men and women, but Kabatereine et al. [[11]] in Uganda found that men are more aware of the disease. In the Volta basin of Ghana, Yirenya-Tawiah et al. [[16]] found that men were more likely to identify the Volta river as the source of infection. Rassi et al. [[31]] in Mozambique found that men were more knowledgeable than women, and Dawaki et al. [[32]] concluded that age <18 years, male sex, increased education, and previous infection are significantly associated with UGS knowledge, but again, none of these findings are directly comparable with ours or with each other because different knowledge assessment tools were used in each, and the study populations were dissimilar.

We chose not to create categories for student scores such as “fair”, “poor”, “good”, etc. because the more relevant component is the specific information that children either possess or lack. For example, while most students connect swimming with UGS, they do not necessarily associate it with other water-related activities such as washing clothes or fetching water; this lack of association makes it unlikely that individuals will consciously engage in preventative behaviors, and presents an opportunity for control programs to better emphasize the risk of all types of skin contact with surface water. As a second example, >80% of students knew that UGS is treatable, but <1% named praziquantel. Knowing the name of the drug to treat UGS is important for several reasons. First, children, their caretakers, and their teachers should know and understand which drugs are distributed through preventative chemotherapy campaigns and how often they are given. Second, praziquantel should be given presumptively at clinics based on symptoms [[33]], and if the clinician does not immediately offer it, parents and children may wish to request it. Third, it is possible to buy praziquantel in Ghana without a prescription, and for families who wish to do so, knowing the name of the drug is key.

Gaps in knowledge have been documented elsewhere with various types of assessments. Studies in the Middle East and sub-Saharan Africa have shown that while people adequately recognize hematuria as a symptom, knowledge about transmission is poor and misconceptions are common [[9], [10], [11], [12], [13], [14], [15], [16], [17], [18], [34]]. Studies have also shown persistent knowledge gaps about risky water contact behaviors [[10], [11], [18], [32], [35]]. In a variety of settings, UGS treatment coverage has been low, and many people do not seek treatment because UGS is perceived as unthreatening, symptoms are perceived as ‘normal’, or treatment costs are assumed to be high [[12], [13], [15], [16], [17], [36]], while some treat the disease with herbal or traditional practices [[12], [17]]. These earlier studies show why knowledge should be systematically assessed and specific gaps should be addressed.

Students in Ghana usually receive UGS lessons from their P3 teachers (not P4), but nevertheless, we found a weak correlation between P4 boys and their teachers’ knowledge. The lack of correlation between JHS2 students and their teachers is puzzling, since they receive UGS lessons from those teachers. However, JHS2 science teachers face a challenge because existing curricular materials contain little or incorrect information about UGS. For example, JHS2 curricula from Kuano, Akyeansa, Nankese, Osorase, and Anamase all incorrectly cite boiling and filtering drinking water as methods of preventing schistosomiasis, which was not the case in other schools. All of these curricula also instruct students to avoid bathing in contaminated water, but do not define what “contaminated water” means, nor do they mention other risky activities that involve skin contact with contaminated water, such as swimming or collecting water for domestic use. Many of the symptoms that teachers mentioned, such as abdominal pain and infertility, are uncommon [[1]], but were frequently mentioned in JHS2 textbooks. None of the JHS2 textbooks we reviewed contained the message that skin contact with water is the mechanism of *S*. *haematobium* transmission. Curricular materials could be improved to emphasize disease transmission, protective measures, and information about praziquantel, and should remove references to drinking water as a transmission method.

Our survey represents a first attempt at a standardized tool to assess UGS knowledge. We reviewed multiple studies that assessed knowledge in some way [[20], [37], [38], [21], [22], [36]] but information about the knowledge assessment was missing and no standardized tools were used or described in sufficient detail to ensure replicability.

### Generalizability

Use of our survey tool at the national level in Ghana and in other endemic countries could provide critical information about ways in which schistosomiasis knowledge is uneven and can be improved through educational and public health initiatives. In the future, the survey tool also could be used in combination with other data to robustly identify the differential roles of demographics, location, socioeconomic status, UGS prevalence, and school instructional techniques, and to prioritize educational interventions accordingly. A future study that collects data about attitudes, practices, and infection status in addition to knowledge would also be relevant.

## Supporting information

S1 Appendix Student Written Knowledge Survey Tool. (DOCX)

S2 Appendix Teacher Written Knowledge Survey Tool. (DOCX)

S1 Table Kolmogorov-Smirnov and Shapiro-Wilks tests to test for departures from normality. (XLSX)

S2 Table Distribution of scores across district, class year, and gender for all students. (XLSX)

S3 Table Distribution of scores across town and class year for all students. (XLSX)

S4 Table Univariate linear regression models (p<0.0005); outcome variable is performance on a UGS knowledge survey administered to 1,813 schoolchildren in the Eastern Region of Ghana using gender (model 1) and class year (model 2) as independent variables. (XLSX)

S5 Table Univariate linear regression models 3 and 4 (p<0.0005); outcome variable is performance on a UGS knowledge survey administered to 1,813 schoolchildren in the Eastern Region of Ghana using district of residence (model 3) and town of residence (model 4) as independent variables. (XLSX)

S6 Table Univariate linear regression models where the outcome variable is UGS knowledge survey score for schoolchildren in the Eastern Region of Ghana and independent variables are teacher performance listing 5 facts about UGS (model 5), overall teacher score on 20 questions that matched student questions (model 6), and P4 (model 7) and JHS2 (model 8) teacher scores with the same 20 questions. (XLSX)

S1 Fig Distribution of student and teacher knowledge scores. Distribution of the overall distribution of knowledge scores both students and teachers with a normal curve overlay.
(TIF)

S2 Fig Boxplots of knowledge scores. Boxplot showing the overall distribution of knowledge scores for boys and girls, broken down by class year.
(TIF)
