# Supplementary material for: Assessment of urogenital schistosomiasis knowledge among primary and junior high school students in the Eastern Region of Ghana: A cross-sectional study
Source: PLoS One. 2019 Jun 13;14(6):e0218080. doi: 10.1371/journal.pone.0218080 (PMC6563970; doi:10.1371/journal.pone.0218080)
Supplement: S1 Appendix — (DOCX) [file pone.0218080.s001.docx]

**Bloody pee Survey**

Are you a BOY or GIRL

What is your age?

What is your year in school?

**How to get bloody pee**

1. If you chew cola nuts you can get bloody pee

true/false/I don’t know

2. If you fetch river or pond water you can get bloody pee

true/false/I don’t know

3. If you bathe with water from a river or pond you can get bloody pee

true/false/I don’t know

4. A curse from another person is one way to get bloody pee

true/false/I don’t know

5. If you swim in a river or pond you can get bloody pee

true/false/I don’t know

6. If you wash a car with river or pond water you can get bloody pee

true/false/I don’t know

7. If you wash clothes or utensils with river or pond water you can get bloody pee

true/false/I don’t know

8. A fetish priest can give a person bloody pee

true/false/I don’t know

9. If you use borehole water you can get bloody pee

true/false/I don’t know

**Treatment for bloody pee**

10. Is there a treatment for bloody pee?

yes/no/I don’t know

11. What is the name of the treatment for bloody pee?

12. If you get bloody pee and it goes away, can you get bloody pee again?

yes/no/I don’t know

**How to protect against bloody pee**

13. I can protect myself against bloody pee by sleeping under a mosquito net.

true/false/I don’t know

14. I can protect myself against bloody pee by not swimming in a river or pond.

true/false/I don’t know

15. I can protect myself against bloody pee by not bathing with water from a river or pond.

true/false/I don’t know

16. I can protect myself against bloody pee by always wearing shoes outside.

true/false/I don’t know

**Symptoms of bloody pee**

17. If I have bloody pee I will see red urine

True/false/I don’t know

18. If I have bloody pee I will see blood in my urine

True/false/I don’t know

19. If I have bloody pee I will have painful urination

True/false/I don’t know

20. If I have bloody pee I will have a fever and chills

True/false/I don’t know

21. Snails in a river or pond can give you bloody pee

True/false/I don’t know

22. It is fine to urinate in the river

True/false/I don’t know
