# Supplementary material for: Assessment of urogenital schistosomiasis knowledge among primary and junior high school students in the Eastern Region of Ghana: A cross-sectional study
Source: PLoS One. 2019 Jun 13;14(6):e0218080. doi: 10.1371/journal.pone.0218080 (PMC6563970; doi:10.1371/journal.pone.0218080)
Supplement: S2 Appendix — (DOCX) [file pone.0218080.s002.docx]

**Teacher Written Knowledge Survey**

Teachers answered “yes” or “no”, rather than “true” or “false” because their survey questions were formatted slightly differently from the student questions. The student survey needed to make sense in the context of a spoken Twi translation, while the teacher survey did not.

**Bilharzia Survey**

Are you MALE or FEMALE

1. List five facts that you know about bilharzia

2. Is there is a treatment for bilharzia?

yes/no

3. If you answered yes to question 3, what is the name of the treatment?

1. Is bilharzia in your syllabus?

yes/no

2. Skip this question if you answered yes to question one. If bilharzia is not in your syllabus have you ever taught your students about bilharzia from your own knowledge?

yes/no

3. Chewing cola nuts is one way to get bilharzia

yes/no

4. Fetching river or pond water is one way to get bilharzia

yes/no

5. Drinking river or pond water is one way to get bilharzia

yes/no

6. Drinking dirty water is one way to get bilharzia

yes/no

7. Using a dirty latrine is one way to get bilharzia

yes/no

8. Fishing in a river is one way to get bilharzia

yes/no

9. Bathing in a river or pond is one way to get bilharzia

yes/no

10. Exercising too hard is one way to get bilharzia

yes/no

11. Eating spoiled food is one way to get bilharzia

yes/no

12. A curse from another person is one way to get bilharzia

yes/no

13. Swimming in a river or pond is one way to get bilharzia

yes/no

14. Washing a car with river or pond water is one way to get bilharzia

yes/no

15. Crossing a river or pond is one way to get bilharzia

yes/no

16. A fetish priest can give a person bilharzia

yes/no

17. Touching the river when heavy rains have made it dirty is one way to get bilharzia

yes/no

18. Being a gold miner is one way to get bilharzia

yes/no

19. Splashing in the river is one way to get bilharzia

yes/no

20. If you get bilharzia and it goes away, can you get bilharzia again?

yes/no

21. The treatment for bilharzia protects against reinfection for:

1. it does not protect against reinfection
2. 24 hours
3. One week
4. One month

22. The treatment for bilharzia is called:

a. Mebendazole

b. praziquantel

c. ivermectin

d. albendazole

23. You can protect yourself against bilharzia by sleeping under a mosquito net.

yes/no

24. You can protect yourself against bilharzia by not swimming in a river or pond.

yes/no

25. You can protect yourself against bilharzia by wearing rubber boots while fishing or crossing streams.

yes/no

26. You can protect yourself against bilharzia by fetching water from boreholes instead of the river.

yes/no

27. You can protect yourself against bilharzia by only drinking pure water.

yes/no

28. You can protect yourself against bilharzia by not bathing in the river or pond.

yes/no

29. You can protect yourself against bilharzia by washing with soap after swimming in the river or pond.

yes/no

30. You can protect yourself against bilharzia by always wearing shoes outside.

yes/no

**Risk factor: An action or exposure that increases the chance of getting an infection**

31. Contact with river or pond water is a risk factor for bilharzia

yes/no

32. Urination in river or pond water is a risk factor for bilharzia

yes/no

33. Using a dirty latrine is a risk factor for bilharzia

yes/no

34. Drinking dirty water is a risk factor for bilharzia

yes/no

35. Getting bitten by a mosquito is a risk factor for bilharzia

yes/no

36. Women fetching water during menstruation is a risk factor for bilharzia

yes/no

37. Touching someone with bilharzia is a risk factor for bilharzia

yes/no

38. If you have bilharzia you will see red urine

Yes/no

39. If you have bilharzia you will see blood in your urine

Yes/no

40. If you have bilharzia you will have painful urination

Yes/no

41. If you have bilharzia you will have fever and chills

yes/no

42. If you think that you have bilharzia, you should (circle all that are true – it’s okay to circle more than one answer):

1. Tell a parent
2. Tell a teacher
3. Go to a pharmacy
4. Go to a clinic
5. Wait for it to go away

43. Snails play an important role in bilharzia

Yes/no

44. The river is a good place to fetch drinking water.

Yes/no

45. A hand-dug well is a good place to fetch drinking water.

Yes/no

46. The river is a good source of bathing water.

Yes/no

47. A shallow well is a good source of bathing water.

Yes/no

48. It is fine to urinate in the river.

yes/no

49. What is the best strategy you would tell your students to use to avoid bilharzia? (For example, the best strategy to use to avoid malaria is sleeping under a mosquito net)
