# Supplementary figures and images for: Assessment of urogenital schistosomiasis knowledge among primary and junior high school students in the Eastern Region of Ghana: A cross-sectional study
Source: PLoS One. 2019 Jun 13;14(6):e0218080. doi: 10.1371/journal.pone.0218080 (PMC6563970; doi:10.1371/journal.pone.0218080)

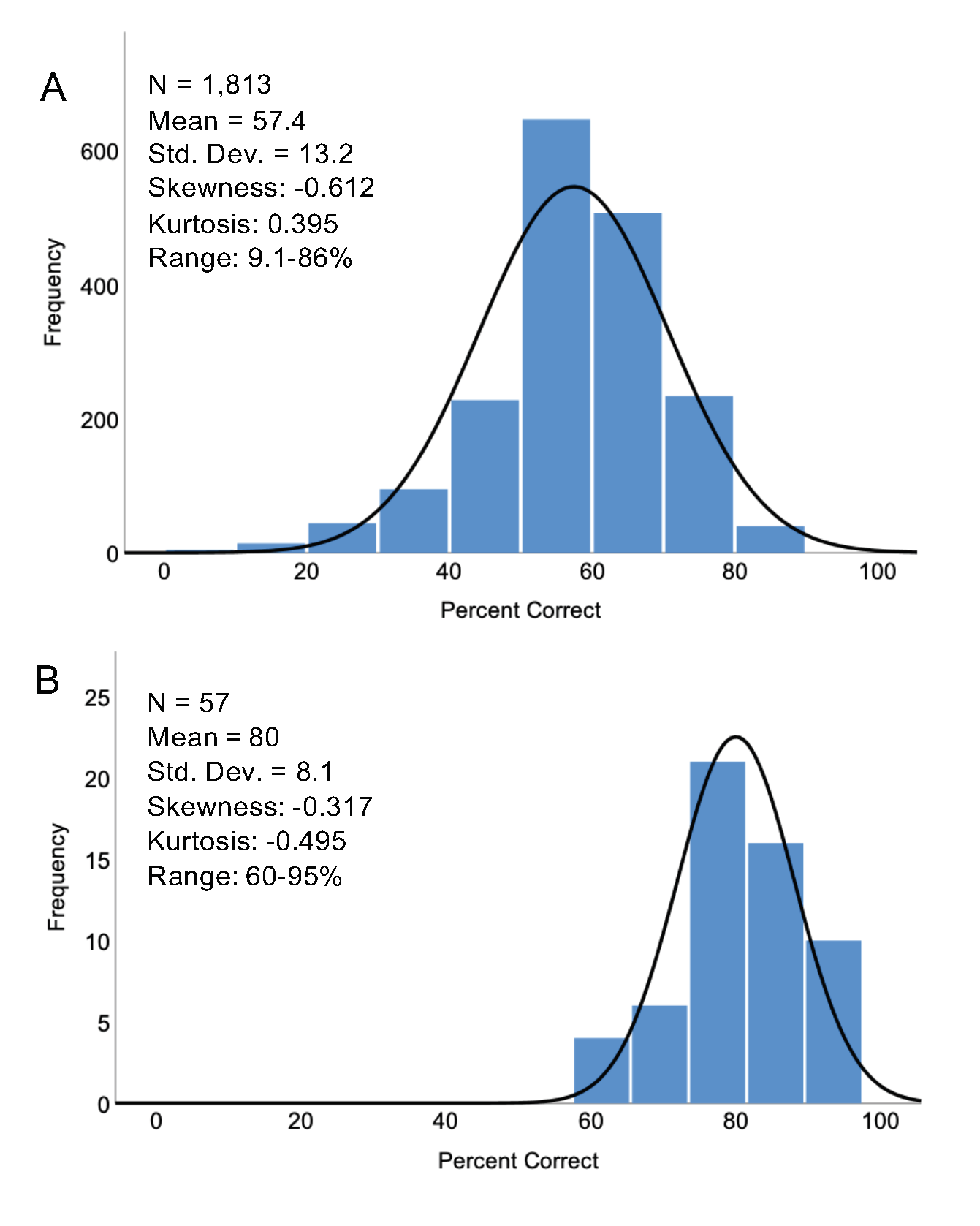

Supplement: S1 Fig — Distribution of the overall distribution of knowledge scores both students and teachers with a normal curve overlay. (TIF) [file pone.0218080.s009.tif]

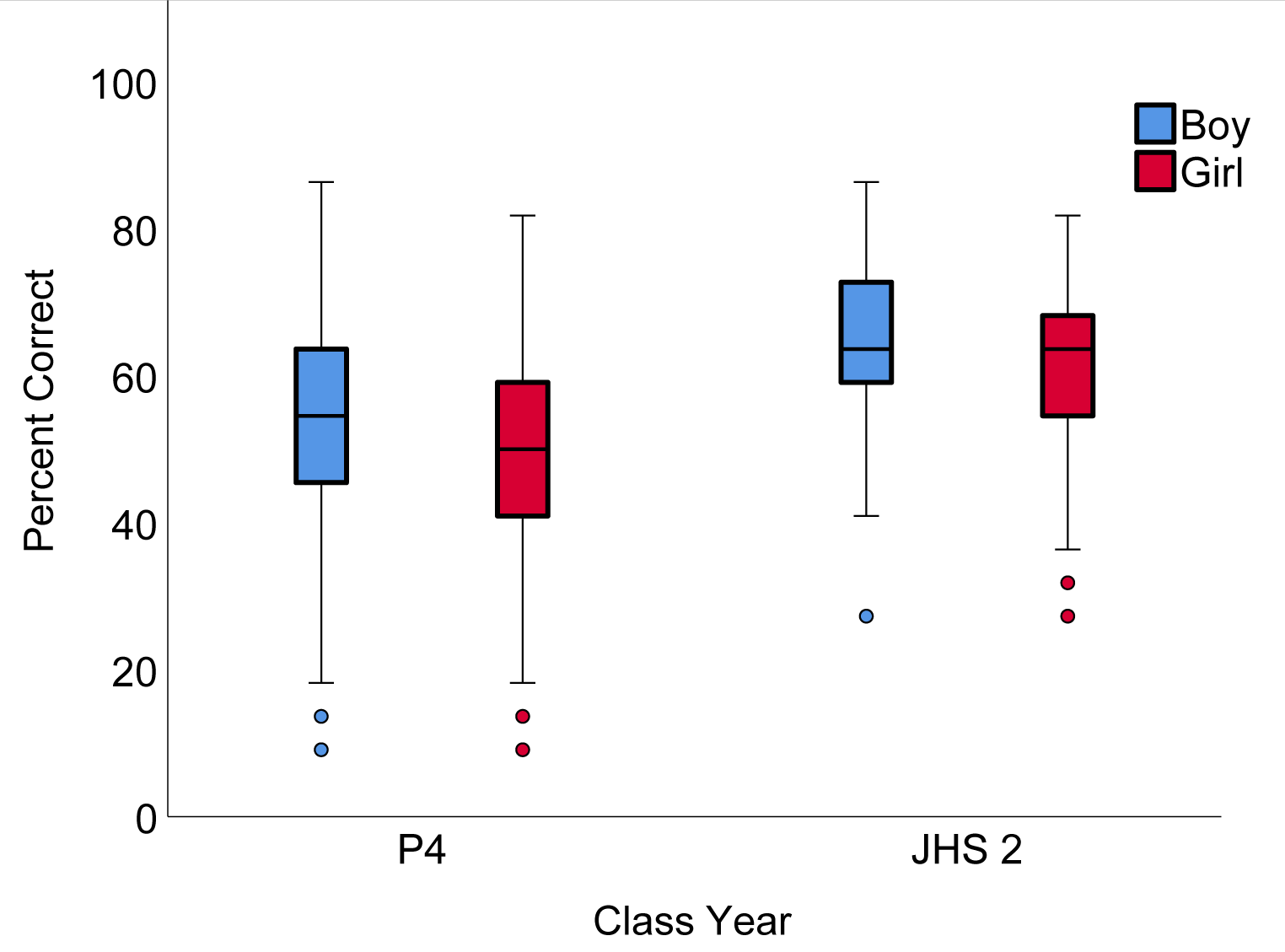

Supplement: S2 Fig — Boxplot showing the overall distribution of knowledge scores for boys and girls, broken down by class year. (TIF) [file pone.0218080.s010.tif]
